# Supplementary figures and images for: Using contextual factors to elicit placebo and nocebo effects: An online survey of healthcare providers’ practice
Source: PLoS One. 2023 Sep 1;18(9):e0291079. doi: 10.1371/journal.pone.0291079 (PMC10473518; doi:10.1371/journal.pone.0291079)

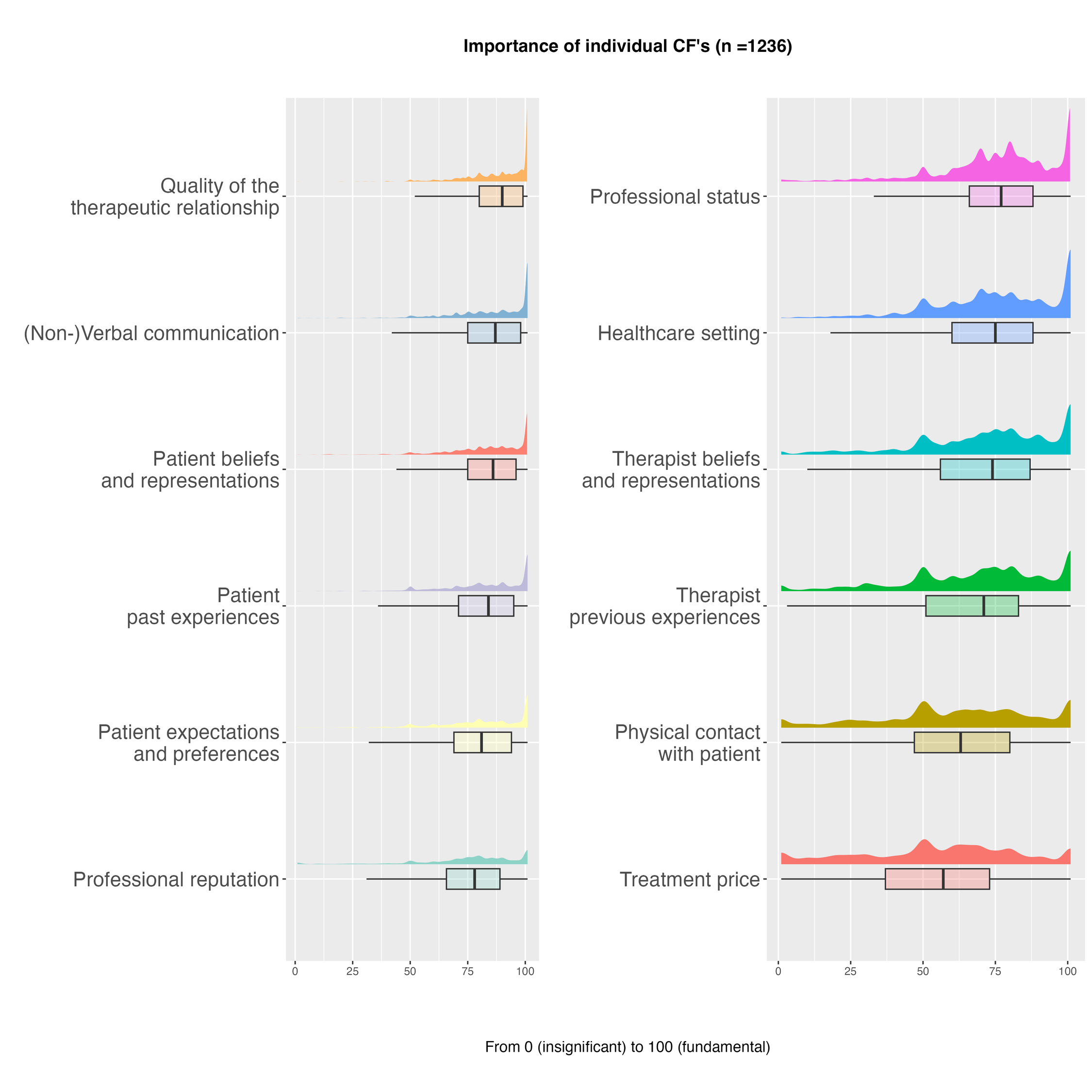

Supplement: S1 Fig — (TIF) [file pone.0291079.s001.tif]

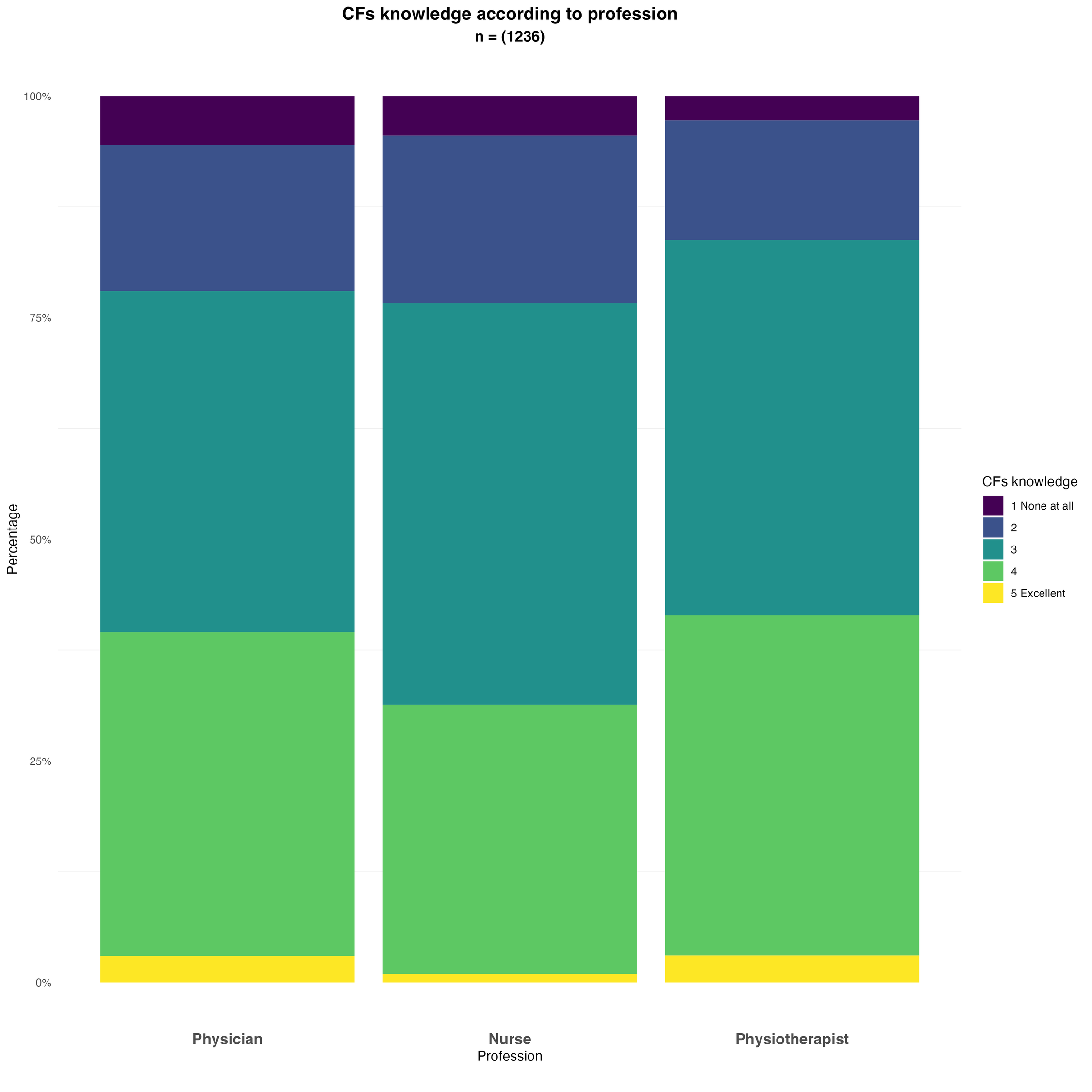

Supplement: S2 Fig — (TIF) [file pone.0291079.s002.tif]

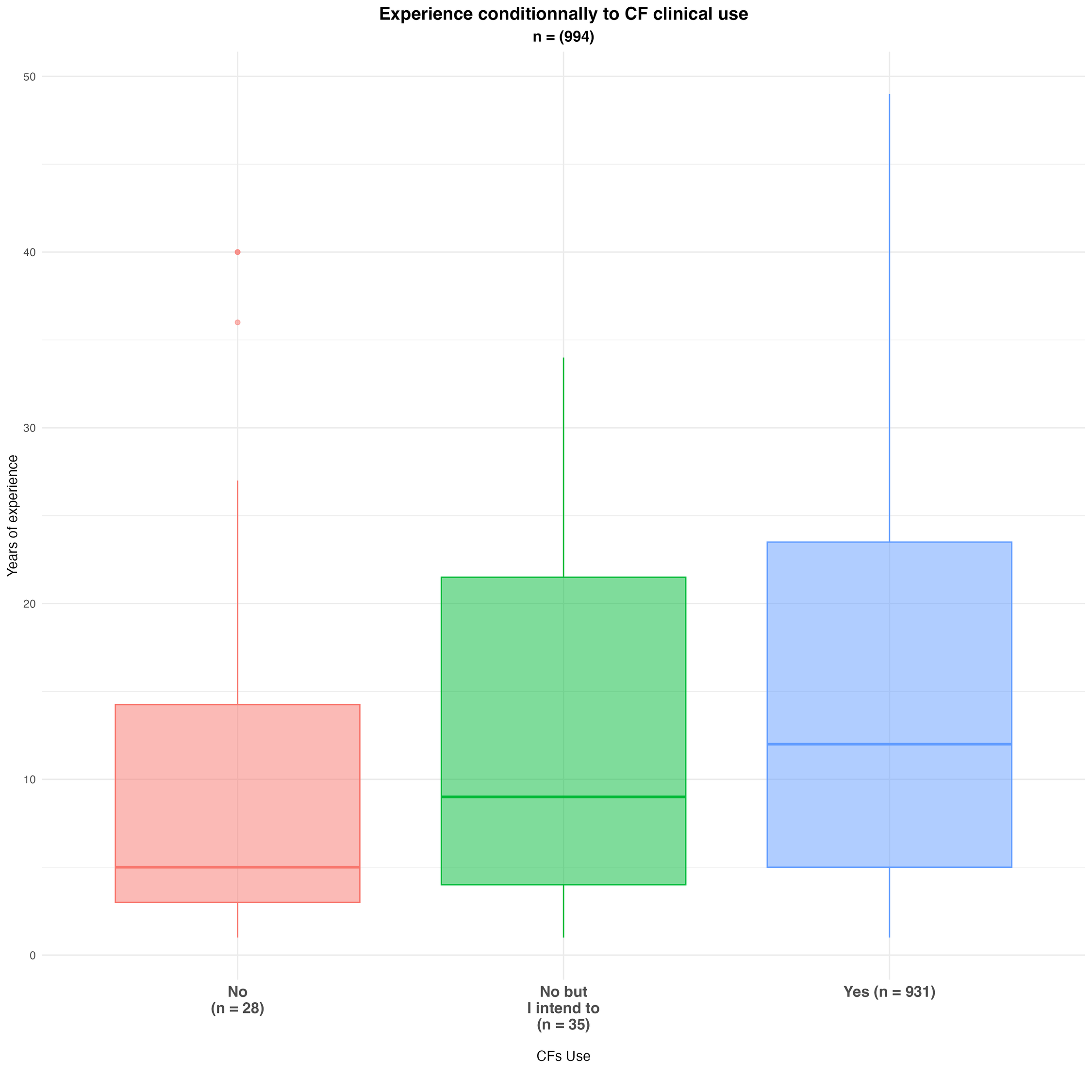

Supplement: S3 Fig — (TIF) [file pone.0291079.s003.tif]

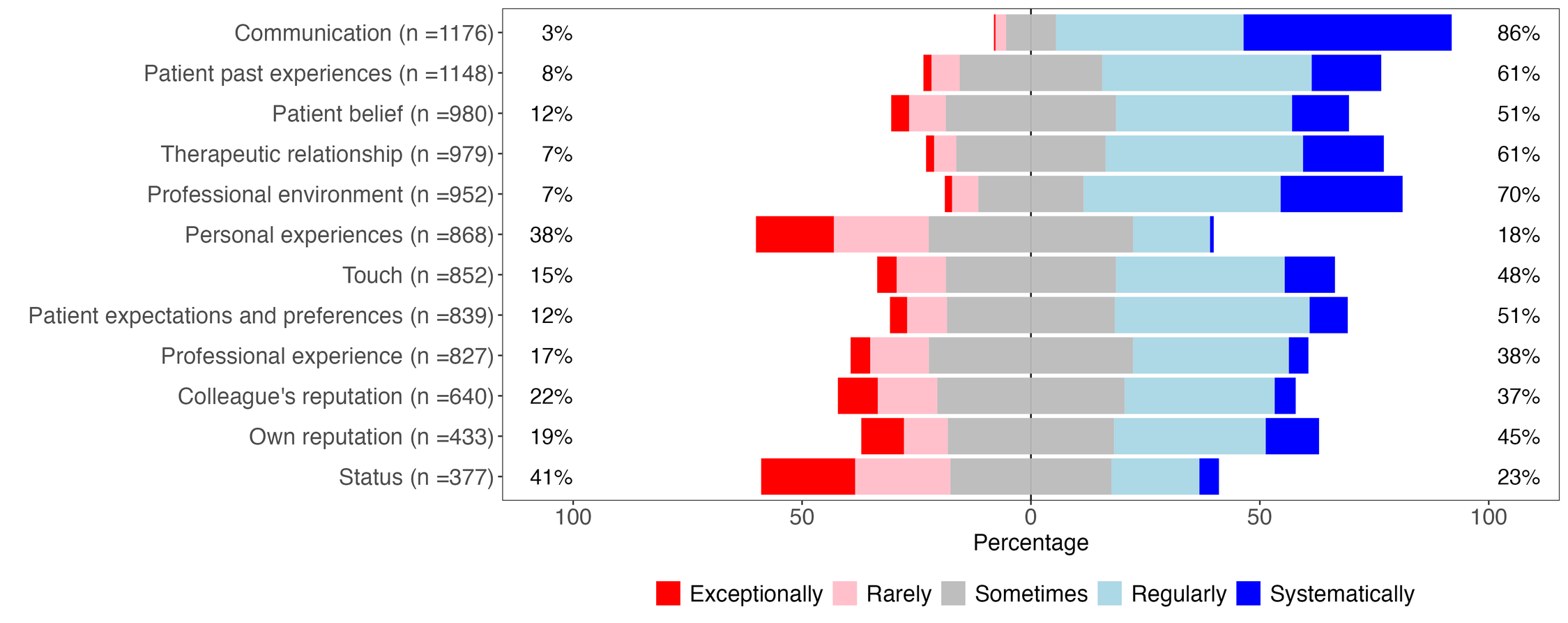

Supplement: S4 Fig — (TIF) [file pone.0291079.s004.tif]

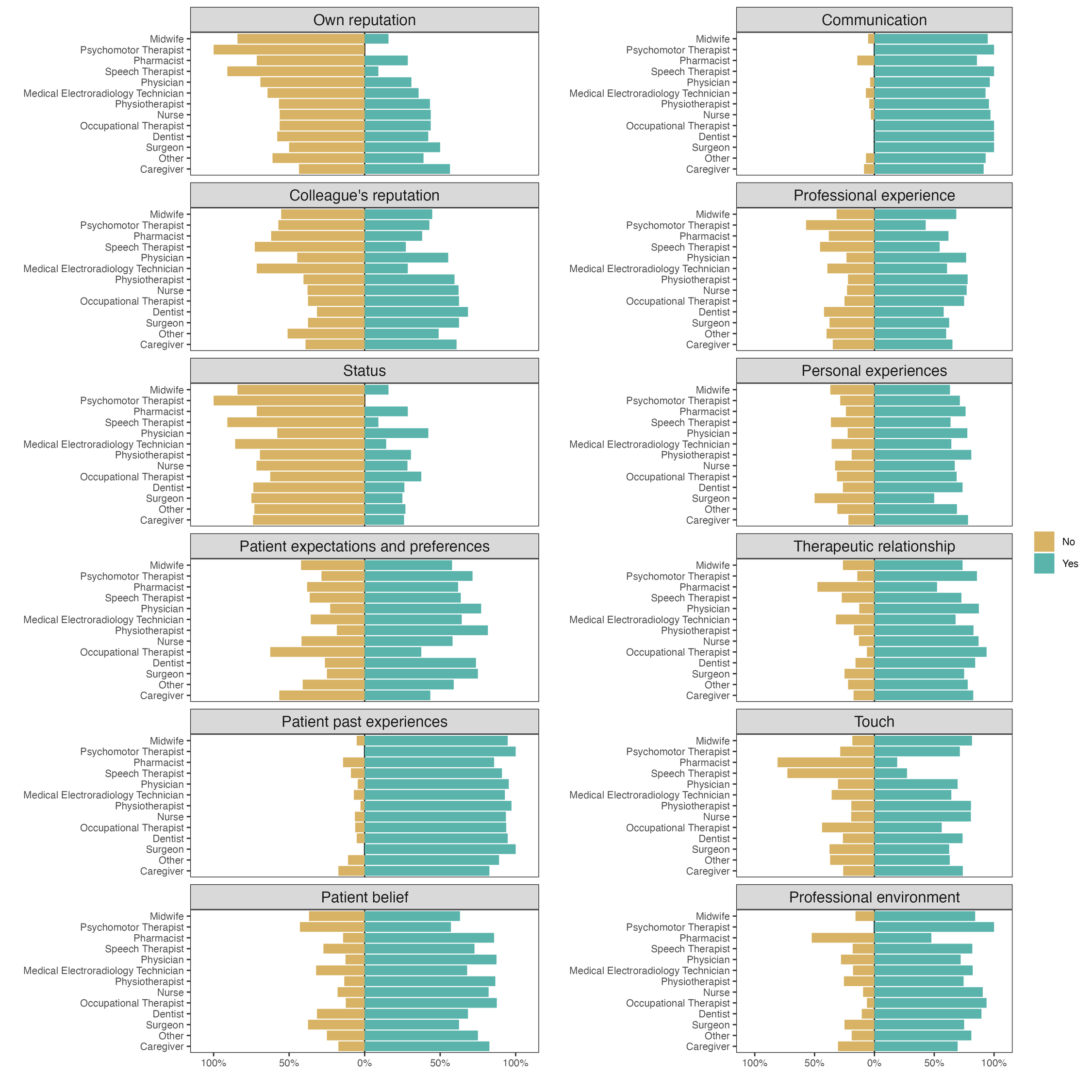

Supplement: S5 Fig — (TIF) [file pone.0291079.s005.tif]
