# Supplementary material for: Using contextual factors to elicit placebo and nocebo effects: An online survey of healthcare providers’ practice
Source: PLoS One. 2023 Sep 1;18(9):e0291079. doi: 10.1371/journal.pone.0291079 (PMC10473518; doi:10.1371/journal.pone.0291079)
Supplement: S1 Table — (DOCX) [file pone.0291079.s007.docx]

| Importance of individual contextual factors | Mean (%) | SD |
| --- | --- | --- |
| Quality of the therapeutic relationship | 87.2 | 13.8 |
| Verbal and non-verbal communication | 83.5 | 16.8 |
| Patient beliefs and representations on symptoms | 83.0 | 16.4 |
| Patient expectations and preferences | 81.0 | 17.3 |
| Patient past-experiences | 78.7 | 19.0 |
| Professional status | 74.4 | 19.7 |
| Professional reputation | 74.2 | 21.1 |
| Healthcare setting | 72.8 | 20.1 |
| Therapist beliefs and representations | 70.0 | 22.6 |
| Therapist previous experiences | 66.9 | 23.2 |
| Physical contact with patient | 60.7 | 24.9 |
| Treatment price | 54.6 | 25.3 |
