# Supplementary material for: Using contextual factors to elicit placebo and nocebo effects: An online survey of healthcare providers’ practice
Source: PLoS One. 2023 Sep 1;18(9):e0291079. doi: 10.1371/journal.pone.0291079 (PMC10473518; doi:10.1371/journal.pone.0291079)
Supplement: S2 Table — (DOCX) [file pone.0291079.s008.docx]

| Proportion of effect attributable to contextual factors | Mean | SD |
| --- | --- | --- |
| Overall | 51.5 | 17.6 |
| In women | 51.9 | 18.7 |
| In men | 49.3 | 18.7 |
| In children | 61.3 | 22.3 |
| In adults | 52.4 | 18.7 |
| In older adults | 57.6 | 21.0 |
| For subjective symptoms | 66.9 | 19.1 |
| For objective symptoms | 43.0 | 21.9 |
