# Supplementary material for: Using contextual factors to elicit placebo and nocebo effects: An online survey of healthcare providers’ practice
Source: PLoS One. 2023 Sep 1;18(9):e0291079. doi: 10.1371/journal.pone.0291079 (PMC10473518; doi:10.1371/journal.pone.0291079)
Supplement: S1 Appendix — (PDF) [file pone.0291079.s009.pdf]

Introduction

Bienvenue dans cette enquête !

Chers consœurs, confrères, étudiantes, étudiants,

Cette enquête vise à étudier dans quelle mesure les professionnel(le)s de santé utilisent le contexte dans lesquels ils prennent en charge leurs patients. Celui-ci peut améliorer ou détériorer le résultat des traitements qu'ils administrent.
En effet, il est admis que des facteurs en lien avec la relation soignant-soigné ou l'environnement peuvent influencer les résultats des traitements : on les nomme les facteurs contextuels. Par exemple, les termes utilisés pour s'adresser au patient, la posture adoptée par le professionnel ou le fait de porter une blouse peuvent influencer la perception de la douleur, le niveau d'anxiété ou la performance physique d'un patient.

Les professionnels de santé en exercice en France, en Suisse et en Belgique comme les étudiants des filières de santé en formation en France, Suisse ou Belgique peuvent répondre à ce questionnaire.

Dans les questions qui suivent, **nous vous demandons de bien vouloir répondre <u>en fonction de votre propre expérience</u>**.

Votre participation à l'étude nécessite 10 à 15 minutes et votre contribution ne sera enregistrée que si l'ensemble du questionnaire est complété. Ainsi, si vous choisissez de ne pas aller au bout du remplissage du questionnaire, aucune donnée ne sera enregistrée. La participation à cette étude ne présente aucun risque ou bénéfice direct pour les participant(e)s. À cet égard, cette étude a fait l'objet d'une déclaration auprès du Comité d'Éthique de la Recherche de l'Université Grenoble Alpes.
Les réponses sont pseudo-anonymes et ne seront utilisées qu'à des fins de recherche. Les données produites sont stockées et traitées dans le respect du RGPD sur des serveurs de l'Université Grenoble Alpes conformes aux exigences du RGPD. Le traitement des données est conforme avec une méthodologie de référence (MR004) de la CNIL.

En cliquant sur "Suivant", vous acceptez de participer à l'étude et consentez à l'utilisation des données produites par la réponse à ce questionnaire dans les conditions décrites plus haut. Les résultats de cette recherche-ci feront l'objet d'une publication scientifique ainsi que de présentations en congrès. En aucun cas, l'anonymat de la participation ne peut être levé et les résultats ne seront présentés que de manière groupée.

Si au terme de l'étude vous souhaitez retirer votre participation ou que vous souhaitez obtenir un renseignement, vous pouvez contacter le coordinateur de l'étude : leo.druart@univ-grenoble-alpes.fr.

Autoévaluation des connaissances

D'après vous, quel est l'état de vos connaissances sur les effets contextuels :

Pas de connaissances — Excellente connaissance du sujet

D'après vous, est-ce que ces connaissances influencent votre pratique clinique ?

Pas du tout — Beaucoup

D'après vous, l'effet contextuel est défini comme :

- Je ne sais pas
- Un traitement n'ayant pas d'efficacité propre ou spécifique
- Aucune des propositions
- Une manifestation des différents symptômes d'une maladie et de leur évolution au cours du temps en l'absence de traitement
- Un effet de l'interaction ou de la présence avec un(e) soignant(e)
- Un effet psycho-physiologique positif (bénéfique) ou négatif (dommageable) observé après un soin quel qu'il soit

L'ordre des items est aléatoire

Connaissances générales

Dans la suite du questionnaire nous considérerons l'effet contextuel comme étant un effet psycho-physiologique positif (bénéfique) ou négatif (dommageable) observé après un soin quel qu'il soit. Celui-ci peut améliorer ou détériorer le résultat des traitements administrés. En effet, il est admis que certains éléments du contexte peuvent influencer les résultats des traitements : on les nomme les facteurs contextuels. Le langage courant associe souvent le terme effet placebo à ce qui est ici défini comme effet contextuel.

D’après vous, les effets contextuels dépendent fortement des paramètres suivants (plusieurs options possibles) :

- ☐ Les caractéristiques du traitement (nature, durée, mode d'administration, durée, etc)
- ☐ Les caractéristiques du patient
- ☐ Les caractéristiques du thérapeute
- ☐ Les caractéristiques de l'environnement de soin
- ☐ Les caractéristiques de la relation thérapeutique
- ☐ Aucune des propositions
- ☐ Je ne sais pas

Sélectionnez, parmi ces situations spécifiques, celle(s) où les effets contextuels sont présents :

- ☐ Lorsque le patient prends un traitement sans avis, ni interaction avec un professionnel de santé (i.e. automédication)
- ☐ Lorsque le traitement n’est pas médicamenteux
- ☐ Lorsque la consultation ne conduit pas à un traitement
- ☐ Lorsque la consultation a lieu au domicile du patient
- ☐ Lorsque la consultation a lieu en télé-soin
- ☐ Aucune des propositions
- ☐ Je ne sais pas

Fonctionnement des Effets Contextuels

D’après vous, quelles sont les explications des mécanismes d’action des effets contextuels ?

- [ ] Histoire naturelle de la maladie
- [ ] Auto guérison (mécanisme basé sur la relation corps-esprit)
- [ ] Mécanismes psychologiques
- [ ] Suggestion verbale ou non-verbale
- [ ] Conditionnement
- [ ] Mécanismes biologiques
- [ ] Entités immatérielles (énergies, spiritualité, etc.)
- [ ] Je ne sais pas
- [ ] Autres

Importance de facteurs contextuels

Évaluez l'importance des facteurs suivants sur les effets contextuels (positifs ou négatifs) :

	Négligeable — Fondamental
Statut professionnel et rôle (étudiant, interne, chef de service, spécialiste, etc.)	
Réputation dans sa profession	
Prix du traitement restant à charge (dépassement d'honoraires, actes hors nomenclature, etc.)	
Attentes et préférences du ou de la patient(e)	
Expériences passées des patient(e)s	
Croyances ou représentations du ou de la patient(e) sur sa pathologies, son thérapeute, son traitement	
Communication verbale ou non-verbale	
Qualité de la relation de soin (attitude générale du / de la professionnel(le))	
Expériences passées du ou de la soignant(e)	
Croyances et représentations du ou de la soignant(e) sur la pathologie, le ou la patient(e), le traitement	
Contact physique avec le ou la patient(e)	
Environnement de consultation (confort d’installation, tenue de travail, lieu de prise en charge, etc.)	

Utilisation des facteurs contextuels

Avez-vous déjà mis en place des stratégies pour valoriser ou influencer votre réputation professionnelle dans le but d'améliorer le résultat clinique de vos prises en charge ?

- Oui
- Non

À quelle fréquence ?

Avez-vous déjà mis en place des stratégies pour valoriser ou influencer la réputation professionnelle d'un(e) confrère dans le but d'améliorer le résultat clinique de vos prises en charge ?

- Oui
- Non

À quelle fréquence ?

Avez-vous déjà usé de titres ou d'un statut (étudiant, interne, docteur, professeur, chef de service, spécialiste, etc.), réels ou non, dans le but d'améliorer le résultat clinique de vos prises en charge ?

- Oui
- Non

À quelle fréquence ?

Avez-vous mis en place des stratégies pour influencer les attentes et préférences du ou de la patient(e) dans le but d'améliorer les résultats cliniques de vos prises en charge ?

- Oui
- Non

À quelle fréquence ?

Avez-vous déjà modifié vos prises en charge en fonction des expériences passées du ou de la patient(e) ?

- Oui
- Non

À quelle fréquence ?

Avez-vous déjà modifié votre prise en charge en fonction des croyances ou représentations du ou de la patient(e) sur sa pathologie, son thérapeute, son traitement ?

- Oui
- Non

À quelle fréquence ?

Avez-vous déjà mis en place des stratégies pour adapter votre communication verbale et/ou non-verbale dans le but d'améliorer les résultats cliniques de vos prises en charge ?

- Oui
- Non

À quelle fréquence ?

Avez-vous déjà mis en avant votre expérience professionnelle dans le but d'améliorer le résultat clinique de vos prises en charge ?

- Oui
- Non

À quelle fréquence ?

Avez-vous déjà mis en avant votre vécu personnel dans le but d'améliorer le résultat clinique de vos prises en charge ?

- Oui
- Non

À quelle fréquence ?

Avez-vous déjà mis en place une stratégie d'adaptation de votre relation de soin dans le but d'améliorer le résultat clinique de votre prise en charge ?

- Oui
- Non

À quelle fréquence ?

Avez-vous déjà utilisé le contact physique lors d'un examen ou d'un traitement aux seules fins d'améliorer le résultat clinique de votre prise en charge ?

- Oui
- Non

À quelle fréquence ?

Avez-vous déjà mis en place des stratégies d'adaptation de l'environnement de soin (confort d’installation, tenue de travail, lieu de prise en charge) dans le but d'améliorer le résultat clinique de vos prises en charge ?

- Oui
- Non

À quelle fréquence ?

Perception de l'effet des facteurs contextuels

De manière générale, quelle que soit la prise en charge, les facteurs contextuels expliquent à eux-seuls :

0% de l'amélioration clinique — 100% de l'amélioration clinique

Lorsque la prise en charge concerne des femmes, les facteurs contextuels expliquent à eux-seuls :

0% de l'amélioration clinique — 100% de l'amélioration clinique

Lorsque la prise en charge concerne des hommes, les facteurs contextuels expliquent à eux-seuls :

0% de l'amélioration clinique — 100% de l'amélioration clinique

Lorsque la prise en charge concerne des enfants, les facteurs contextuels expliquent à eux-seuls :

0% de l'amélioration clinique — 100% de l'amélioration clinique

Lorsque la prise en charge concerne des adultes, les facteurs contextuels expliquent à eux-seuls :

0% de l'amélioration clinique — 100% de l'amélioration clinique

Lorsque la prise en charge concerne des personnes agées, les facteurs contextuels expliquent à eux-seuls :

0% de l'amélioration clinique — 100% de l'amélioration clinique

Lorsque la prise en charge vise à traiter des symptômes subjectifs (fatigue, anxiété, douleur, etc), les facteurs contextuels expliquent à eux-seuls :

0% de l'amélioration clinique — 100% de l'amélioration clinique

Lorsque la prise en charge vise à traiter des symptômes objectifs (fréquence cardiaque, glycémie, sécrétion de dopamine, saturation en oxygène, etc), les facteurs contextuels expliquent à eux-seuls :

0% de l'amélioration clinique — 100% de l'amélioration clinique

Conditions personnelles d'utilisation

Suite aux éléments du questionnaire précisant la définition (rappelée ci-dessous) des facteurs contextuels, les utilisez-vous ?

- ○ Oui, je les utilise déjà
- ○ Non
- ○ Non mais j'envisage de les utiliser

Dans quel objectifs ?

- ☐ Dans le cadre des prises en charges efficaces dispensés afin d'optimiser les résultats cliniques
- ☐ Pour compenser le manque d'effet d'un traitement sans efficacité démontrée
- ☐ Pour répondre à un besoin d'amélioration de la satisfaction du ou de la patient(e)
- ☐ Lorsque vous êtes dans une impasse thérapeutique
- ☐ Afin de mieux supporter les effets indésirables des traitements efficaces
- ☐ Autres motivations

Rappel de définition :

L'effet contextuel est un effet psycho-physiologique positif (bénéfique) ou négatif (dommageable) observé après un soin quel qu'il soit. Celui-ci peut améliorer ou détériorer le résultat des traitements administrés. En effet, il est admis que certains éléments du contexte peuvent influencer les résultats des traitements : on les nomme les facteurs contextuels. Le langage courant associe souvent le terme effet placebo à ce qui est ici défini comme effet contextuel.

Démographie

Quel est votre genre ?

- Homme
- Femme
- Autre

Quel âge avez-vous ?

Comment avez-vous entendu parler de ce questionnaire ?

- Réseaux Sociaux
- Mailing
- Bouche à oreille
- Autre

Vous exercez / étudiez

- en France métropolitaine
- en France non-métropolitaine
- en Suisse
- en Belgique
- Autre

Vous êtes :

- Professionnel(le)
- Étudiant(e)

Démographie des Professionnels

Quelle est votre profession ?

- Aide-soignant(e)
- Chirurgien
- Dentiste
- Ergothérapeute
- Infirmier(e)
- Kinésithérapeute / Physiothérapeute
- Manipulateur(rice) en électroradiologie médicale
- Médecin
- Orthophoniste
- Orthoptiste
- Pharmacien(ne) d'officine
- Psychomotricien(ne)
- Sage-femme
- Autre (préciser)

Avez-vous une pratique de spécialité ?

- I.P.A.
- I.B.O.D.E.
- I.A.D.E.
- Puericulteur/trice
- Aucun

Depuis combien de temps êtes vous diplômé(e) (en années entières) ?

Quel est votre mode d'exercice clinique principal ?

- ◯ Salarié du secteur public
- ◯ Salarié du secteur privé
- ◯ Libéral ou indépendant
- ◯ Mixte
- ◯ Autre

Quel exercice préférentiels avez-vous ?

☐ Biologie médicale	☐ Cardiologie	☐ Chirurgie
☐ Dermatologie et vénéréologie	☐ Esthétique et reconstruction	☐ Endocrinologie - Maladies métaboliques
☐ Gastro-entérologie et hépatologie	☐ Génétique médicale	☐ Gériatrie
☐ Gynécologie et obstétrique	☐ Hématologie	☐ Maladie infectieuse
☐ Maxillo-faciale	☐ Médecine du travail	☐ Médecine générale
☐ Médecine interne	☐ Médecine nucléaire	☐ Médecine physique et de réadaptation
☐ Néphrologie	☐ Neurologie	☐ Ophtalmologie
☐ ORL	☐ Orthopédie et traumatologie	☐ Oncologie
☐ Pédiatrie	☐ Pneumologie	☐ Psychiatrie
☐ Radiologie et imagerie médicale	☐ Réanimation et anesthésie	☐ Rhumatologie
☐ Santé publique	☐ Urologie	☐ Autres :

Intervenez vous auprès d'une catégorie de population particulière ?

- ◯ Oui
- ◯ Non

Si oui, laquelle ?

- ☐ Nouveaux-nés
- ☐ Nourrissons
- ☐ Adolescents - Enfants
- ☐ Adultes
- ☐ Personnes âgées
- ☐ Patients en fin de vie
- ☐ Patients avec maladies professionnelles
- ☐ Sportifs
- ☐ Patients avec une atteinte cognitive
- ☐ Douleurs persistantes
- ☐ Population précaires
- ☐ Affections de longue durée
- ☐ Autres :

Démographie Étudiants

Vous êtes étudiants :

- En Orthophonie
- En Kinésithérapie / En Physiothérapie
- En Ergothérapie
- En Médecine
- En Maïeutique
- En Pharmacie
- En Manipulation radio
- En Puériculture
- En Aide-soignant(e)
- En Soins infirmiers
- En Odontologie
- En Orthoptie
- En Psychomotricité
- Autres (préciser)

Vous êtes :

- Externe
- Interne de spécialité médicale
- Interne de spécialité chirurgicale

Vous êtes :

- Externe
- Interne de pharmacie d'officine
- Interne de pharmacie d'une autre spécialité

En quelle année êtes-vous de votre parcours (exemple 3ème année depuis le bac hors redoublement, mettre "3") ?
