# Supplementary material for: Using contextual factors to elicit placebo and nocebo effects: An online survey of healthcare providers’ practice
Source: PLoS One. 2023 Sep 1;18(9):e0291079. doi: 10.1371/journal.pone.0291079 (PMC10473518; doi:10.1371/journal.pone.0291079)
Supplement: S2 Appendix — (PDF) [file pone.0291079.s010.pdf]

Introduction

Welcome to this survey !

Dear colleagues and students,

The aim of this survey is to investigate the extent to which health professionals use the context in which they care for their patients. This can improve or worsen the outcome of the treatments they administer.
Indeed, it is recognized that factors related to the provider-patient relationship or the environment can influence treatment outcomes: these are known as contextual factors. For example, the words used to address the patient, the posture adopted by the professional or the fact of wearing a gown can influence the perception of pain, the level of anxiety or the physical performance of a patient.

Practicing health professionals in France, Switzerland and Belgium as well as healthcare students in France, Switzerland or Belgium can answer this questionnaire.

In the following questions, **we ask you to answer according to <u>your own experience</u>**.

Your participation in the survey takes 10-15 minutes and your contribution will only be recorded if the whole questionnaire is completed. Therefore, if you choose not to complete the questionnaire, no data will be recorded. There is no direct risk or benefit to participants in this study. In this respect, this study has been declared to the Research Ethics Committee of Grenoble Alpes University.
The responses are pseudo-anonymous and will only be used for research purposes. The data produced is stored and processed in compliance with the RGPD on Université Grenoble Alpes servers that comply with the RGPD requirements. The data processing complies with a national methodology of reference (MR004) of the CNIL.

By clicking on "Next", you agree to participate in the study and consent to the use of the data produced by the response to this questionnaire under the conditions described above. The results of this research will undergo scientific publication and be presented at conferences. Under no circumstances can the anonymity of participation be lifted and the results will only be presented in an aggregate.

If at the end of the study you wish to withdraw your participation or if you need information, you can contact the study coordinator: EMAIL ADRESS

Knowledge self-evaluation

What do you think is the state of your knowledge about contextual effects?

No knowledge — Excellent knowledge on the topic

In your opinion, does this knowledge influence your clinical practice?

Not at all — A lot

According to you, the contextual effect is defined as :

- A manifestation of symptoms of a condition or their evolution, positive or negative, in absence of treatment
- I don't know
- A treatment with no specific efficacy
- A positive (beneficial) or negative (detrimental) psycho-physiological effect observed after a treatment regardless of its specific efficacy
- An effect of the interaction with a therapist
- None of the following

The order of items is random

General understanding

In the rest of the questionnaire we will consider the contextual effect as being a positive (beneficial) or negative (damaging) psycho-physiological effect observed after any kind of treatment. This can improve or deteriorate the outcome of the treatments administered. Indeed, it is accepted that certain elements of the context can influence the results of treatments: these are called contextual factors. Commonly the term placebo effect is often associated with what is defined here as a contextual effect.

In your opinion, the contextual effects depend strongly on the following parameters (several options possible):

- ☐ Treatment characteristics (type, duration, administration mode, etc)
- ☐ Patient characteristics
- ☐ Therapist characteristics
- ☐ Healthcare environnement setting characteristics
- ☐ Therapeutic relationship characteristics
- ☐ None of the above
- ☐ I don't know

Select the specific situation(s) where contextual effects are present:

- ☐ When the patient takes a treatment without advice from or interaction with a health professional (i.e. self-medication)
- ☐ When the treatment is not medicinal
- ☐ When the consultation does not lead to treatment
- ☐ When the consultation takes place at the patient's home
- ☐ When the consultation takes place via telecare
- ☐ None of the above
- ☐ I don't know

How contextual effects work

What do you think are the explanations for the mechanisms of action of contextual effects?

- [ ] Spontaneous evolution of the disease
- [ ] Self-healing (based of the mind-body relationship)
- [ ] Psychological mechanisms
- [ ] Verbal or non-verbal sugestions
- [ ] Condtionning
- [ ] Biological mechanisms
- [ ] Immaterial entities (energies, spirituality, etc.)
- [ ] I don't know
- [ ] Other

Importance of contextual factors

Assess the importance of the following factors on contextual effects (positive or negative):

	Negligible ... Fundamental
Professional status and role (student, intern, head of department, specialist, etc.)	
Reputation in the profession	
Price of treatment remaining to be paid (additional fees, non-nomenclature procedures, etc.)	
Patient's expectations and preferences	
Past experiences of patients	
Beliefs or representations of the patient about his/her pathology, therapist, treatment	
Verbal or non-verbal communication	
Quality of the care relationship (general attitude of the professional)	
Past experiences of the carer	
Beliefs and representations of the carer about the disease, the patient, the treatment	
Physical contact with the patient	
Consultation environment (comfort of installation, working clothes, place of care, etc.)	

Contextual Factors Use

Have you ever implemented strategies to enhance or influence your professional reputation in order to improve the clinical outcome of your care?

- ○ Yes
- ○ No

How often ?

Have you ever implemented strategies to enhance or influence the professional reputation of a colleague in order to improve the clinical outcome of your care?

- ○ Yes
- ○ No

How often ?

Have you ever used titles or status (student, intern, doctor, professor, head of department, specialist, etc.), real or not, in order to improve the clinical outcome of your care?

- ○ Yes
- ○ No

How often ?

Have you used strategies to influence patient expectations and preferences in order to improve clinical outcomes?

- Yes
- No

How often ?

Have you ever modified your treatments according to the patient's past experiences?

- Yes
- No

How often ?

Have you ever modified your approach according to the patient's beliefs or representations about his or her pathology, therapist or treatment?

- Yes
- No

How often ?

Have you ever implemented strategies to adapt your verbal and/or non-verbal communication in order to improve the clinical outcomes of your care?

- Yes
- No

How often ?

Have you ever put forward your professional experience to improve the clinical outcome of your treatments?

- Yes
- No

How often ?

Have you ever put forward your personal experiences to improve the clinical outcome of your care?

- Yes
- No

How often ?

Have you ever put in place a strategy to adapt your therapeutic relationship in order to improve the clinical outcome of your care?

- Yes
- No

How often ?

Have you ever used physical contact during an examination or treatment for the sole purpose of improving the clinical outcome of your care?

- Yes
- No

How often ?

Have you already implemented strategies for adapting the care environment (comfort of installation, working clothes, place of care) in order to improve the clinical outcome of your care?

- Yes
- No

How often ?

Perception of effect of contextual factors

In general, for all types of care, contextual factors alone account for :

0% of clinical improvement — 100% of clinical improvement

Where care is provided to women, contextual factors alone account for :

0% of clinical improvement — 100% of clinical improvement

Where care is provided to men, contextual factors alone account for :

0% of clinical improvement — 100% of clinical improvement

Where care is provided to children, contextual factors alone account for :

0% of clinical improvement — 100% of clinical improvement

Where care is provided to adults, contextual factors alone account for :

0% of clinical improvement — 100% of clinical improvement

Where care is provided to older people, contextual factors alone account for :

0% of clinical improvement — 100% of clinical improvement

When dealing with subjective symptoms, contextual factors alone account for :

0% of clinical improvement — 100% of clinical improvement

When dealing with objective symptoms, contextual factors alone account for :

0% of clinical improvement — 100% of clinical improvement

Personal terms of use

Following the questionnaire items specifying the definition (recalled below) of contextual factors, do you use them?

- ○ Yes, I already use them
- ○ No
- ○ No but I plan to use them

For what purposes ?

- ☐ In the context of effective treatments provided to optimise clinical outcomes
- ☐ To compensate for the lack of effect of a treatment without proven effectiveness
- ☐ To meet a need to improve patient satisfaction
- ☐ When you are in a therapeutic impasse
- ☐ To better cope with the side effects of effective treatments
- ☐ Other motivations

Reminder of definition :
The contextual effect is a positive (beneficial) or negative (damaging) psycho-physiological effect observed after any treatment. It can improve or deteriorate the outcome of the treatments administered. Indeed, it is accepted that certain elements of the context can influence the results of treatments: these are called contextual factors. Commonly, the term placebo effect is often associated with what is defined here as a contextual effect.

Demography

What is your gender ?

- Man
- Woman
- Other

How old are you ?

How did you hear about this questionnaire ?

- Social media
- Email
- Word of mouth
- Other

You work / study:

- in mainland France
- in non-metropolitan France
- in Switzerland
- in Belgium
- Other

You are a:

- Professional
- Student

Demography for Professionals

What is your profession ?

- Carer
- Surgeon
- Dentist
- Occupational Therapist
- Nurse
- Physiotherapist
- Medical Electroradiology Technician
- Physician
- Speech Therapist
- Orthoptist
- Pharmacist
- Psychomotor therapist
- Midwife
- Autre (préciser)

Do you have a speciality practice ?

- I.P.A.
- I.B.O.D.E.
- I.A.D.E.
- Childcare
- None

How long have you been a graduate (in whole years)?

What is your main clinical practice?

- Employed public sector
- Employed private sector
- Private Practice
- Mixed
- Other

What is your preferred exercise?

- Medical Biology
- Dermatology
- Gastroenterology and hepatology
- Gynaecology and obstetrics
- Maxillofacial
- Internal Medicine
- Nephrology
- ENT
- Pediatrics
- Radiology
- Public Health
- Cardiology
- Plastic Surgery
- Medical Genetics
- Hematology
- Occupational medicine
- Nuclear Medicine
- Neurology
- Orthopaedics and traumatology
- Pneumology
- Resuscitation and anaesthesia
- Urology
- Surgery
- Endocrinology
- Geriatrics
- Infectious diseases
- General Medicine
- Physical and Rehabilitation Medicine
- Ophtalmology
- Oncology
- Psychiatry
- Rhumatology
- Other

Do you work with a particular category of population?

- Yes
- No

If yes, which one ?

- ☐ Newborns
- ☐ Infants
- ☐ Teenagers - Children
- ☐ Adults
- ☐ Older people
- ☐ End of life patients
- ☐ Patients with occupational diseases
- ☐ Sportsmen and women
- ☐ Patients with cognitive impairment
- ☐ Peristant pain
- ☐ Low-income population
- ☐ Long-term conditions
- ☐ Other

Demography for students

You are a student:

- In Speech Therapy
- In Physiotherapy
- In Occupational Therapy
- In Medicine
- Of Maieutics
- In Pharmacy
- In Medical Imagery Techniques
- In Childcare
- to be a carer
- In Nursing
- In Odontology
- In Orthoptics
- In Psychomotor Therapy
- Other

You are:

- Medical Extern
- Medical Specialty Resident
- Surgical Specialty Resident

You are:

- Extern
- Resident in Clinical Pharmacology
- Resident in another Pharmacology Speciliaty

In which year of your course are you (e.g. 3rd year since the baccalaureate without counting repeated years, put "3")?
