# Supplementary material for: Using contextual factors to elicit placebo and nocebo effects: An online survey of healthcare providers’ practice
Source: PLoS One. 2023 Sep 1;18(9):e0291079. doi: 10.1371/journal.pone.0291079 (PMC10473518; doi:10.1371/journal.pone.0291079)
Supplement: S3 Appendix — (PDF) [file pone.0291079.s011.pdf]

# Questionnaire Logic

- All questions are mandatory
- Questions with circle before the items allow for a unique choice and questions with boxes in front of items allow multiple answers
- Questions with answer options such as “Other” allow for free text responses if the item is selected.

<u>Page : Knowledge Self-Evaluation</u>

- Question 2 is only shown when answer to Question 1 is different from “No knowledge”
- Scales range from 1 to 5 with text modalities for variable 1 and 5

<u>Page : Importance of Contextual Factors</u>

- Answers vary on a scale of 1 to 101 with extreme modalities shown as “Negligible” and “Fundamental”

<u>Page : Contextual Factors Use</u>

- Questions regarding pace of use are only displayed in the specific CF is used, i.e. the answer is “Yes”
- Options for frequency of use are “Systematically”; “Regularly”; “Sometimes”; “Rarely”; “Exceptionally” and “I don’t know”.

<u>Page : Perception of effect of contextual factors</u>

- Answers vary from 1 to 101 with text modalities at both extremes shown as “0% of clinical improvement” and “100% of clinical improvement”

<u>Page : Personal terms of use</u>

- The second question “For what purposes” only shows if the answer to the previous question is different from “No”.

<u>Page : Demography</u>

- Depending on the answer to the last question, participants are either directed to the page “Demography for professionals” or “Demography for students”

<u>Page : Demography for Professionals</u>

- The question “Do you have a specialty practice ?” is only shown for nurses
- The question “What is you preferred exercice?” is only shown to physicians and surgeons
- The question “If yes, which one?” is only shown to people having replied “yes” to the previous question

<u>Page : Demography for students</u>

- The first question “You are” is only for medical students
- The second question “You are” is only for pharmacy students
